# Is “Transcolumnar” a better Terminology than “Transverse” in Judet-Letournel Classification of Acetabular Fractures?

**DOI:** 10.5704/MOJ.2107.002

**Published:** 2021-07

**Authors:** A Sood

**Affiliations:** Department of Orthopaedics, International Medical University, Kuala Lumpur, Malaysia

**Keywords:** acetabulum fracture, classification

## Abstract

Acetabular fractures are among the most complex and challenging injuries for orthopaedic surgeons. The choice of surgical treatment in acetabular fracture is important for optimal outcomes. It requires an understanding of the precise outline of the fracture by appropriate classification because it is important for decision making. For this purpose, the classification proposed by Judet and Letournel in 1963 remains the gold standard despite its shortcoming, which are attributed to the inclusion of multiple criteria including anatomical, directional and geometrical. This complex classification remains challenging especially to lesser experienced surgeons. In this article, a new method for classification of acetabulum fractures is proposed. It places posterior column with posterior wall fractures to simple fractures instead of complex fractures in Judet and Letournel classification. More importantly the proposed new classification renames “transverse fracture” as “transcolumnar fracture” to better represent anatomical structure rather than morphology. It is expected that by coining the new term “transcolumnar ”it will be easy to understand different types of acetabular fractures. Especially the confusion between “transverse” and “both column fractures” would not be a concern in future.

## Introduction

Acetabular fractures are injuries that involve a heterogenous group of population. In a previous study, an annual incidence of 37 pelvic fractures per 100,000 population was reported in the USA and Western Europe and 10% of them involved acetabulum^1^. In the USA and Europe, the frequency of fractures was reported to peak in two age groups; the first peak in young patients who sustain high-energy fractures and the second peak among older patients who sustain low-energy osteoporotic fractures^2^. In one of the studies, among 156 senior patients with acetabular fracture and an average age of 77 years, 14% were those aged 65 years or more and 72% of them were male^3^.

Acetabular fractures are among the most complex and challenging injuries for orthopaedic surgeons. The first description of acetabular fractures comes from the poetry of Homer’s Iliad in 8th century BC. Four centuries later, Hippocrates gave a common terminology “hip dislocations” to injuries around the acetabulum as it was impossible to differentiate between hip dislocation from an acetabulum fracture by clinical examination alone. Subsequently greater descriptions of acetabular fractures came to light and the treatment in the first half of 20th century was limited to conservative strategies. In 1963, Judet and Letournel published the classical article to describe fractures of the acetabulum, classification and surgical approaches for open reduction. Their classification enhanced the understanding of complex 3D geometry of the fractures and allowed a logical choice of operative procedures^4^. The concepts proposed by Judet and Letournel took a long time before getting wider acceptance. Later they published two textbooks in 1981 and 1993, which became very popular and are till today considered to be “the bible” for acetabular surgeons. The Judet-Letournel classification proposed in 1964 and refined in 1974 has stood the test of time and remains the most common and practical way of classifying acetabular fractures. However, the shortcomings have been highlighted by many and hence, a newer approach to classify acetabular fracture for better management strategies is of utmost importance. In this article the merits and demerits of the existing classifications are discussed and importantly a newer approach to classify acetabular fractures is proposed.

## Discussion

The choice of surgical treatment in acetabular fracture is important for optimal outcomes and understanding of the precise outline of the fracture is important in decision making. Hence, Judet-Letournel did an extensive study of the anatomy and developed a concept of “two columns” to classify acetabular fractures. Accordingly, three standard radiograph projections (AP, iliac oblique and obturator oblique) and four lines (iliopectineal, ilioischial, two lines representing anterior and posterior walls) are used to understand the 3D morphology of fractures which are classified into five simple and five complex types. Simple fractures include those in which all or a part of one column is fractured (posterior wall, posterior column, anterior wall, anterior column, transverse), whereas complex fractures are those in which at least two of the elementary forms are involved (Table I). This classification helps in pre-operative planning and to determine the most appropriate surgical approach. Studies have shown that intra- and interobserver reliability of the use of this classification is high in an expert group but notably it remains low among less trained surgeons^5,6^. It is well known that this classification is challenging, intimidating and often difficult to understand, especially for beginners. Notably, despite paramount importance of the understanding of the classification of acetabular fractures of appropriate management; the difficulties of lesser trained surgeons were largely attributed to the challenging nature of acetabular fractures, rather than the complexities of the classification system.

**Table I:** Classification of acetabulum fractures as proposed by Judet and Letournel

Simple		Complex	
Name of fracture	Based on	Name of fracture	Based on
Posterior wall	Anatomy	“T” Shape	Geometry*
Posterior column	Anatomy	Transverse with posterior wall	Anatomy Direction*
Anterior wall	Anatomy	Posterior column with posterior wall	Anatomy
Anterior column	Anatomy	Anterior column with Posterior hemi transverse	Anatomy Direction*
Transverse	Direction*	Both Column	Anatomy

Ideally, any classification should be based on a common criterion to determine the type of fractures. If we analyse from this viewpoint, the classification of Judet-Letournel is not based on a common criterion. It takes into account anatomical, morphological and geometrical features, hence making it complex to understand. In view of these shortcoming of the original Judet-Letournel classification, the need for a revision has been realised and many attempts have been made to introduce new classifications based on direction of force, CT scan etc. (including AO comprehensive classification)^7-12^.

In this article, the author makes an attempt to propose a classification which retains the original concept of acetabulum being made of two columns and two walls but is more user friendly and easy to understand. In author’s opinion the introduction of the word “Transverse” is responsible for this ambiguity in Judet-Letournel classification. “Transverse” portrays the “direction” of the fracture. Hence, it represents morphology of fracture and not anatomy, which is the basis of Judet-Letournel classification. This is also the reason for difficulty in understanding and correlating fractures of the same structure with different criteria, e.g., anatomy, direction, and geometry. To address this fallacy, the author proposes the term “Transcolumnar” (anatomical term) instead of “Transverse” (directional). It is also proposed that posterior wall with posterior column fractures to be considered simple fractures to make it more symmetrical.

The proposed modified classification is summarised in (Table II). This suggested classification is still based on the original concept of acetabulum being made of two columns and two walls. However, it is a re-arrangement with introduction of the term “Transcolumnar” instead of “Transverse” to make it symmetrical and easy to comprehend especially for beginners. The new proposed terminology is summarised in (Table III). The author would like to stress that the purpose of this simplification is easier comprehension of the fractures of acetabulum. All the pros and cons of the classification remain the same, with original concept that acetabulum is made of two wall and two columns.

**Table II:** Classification of acetabular fractures as proposed by Author

Simple	Complex
Posterior wall	Transcolumnar Transacetabulum
Posterior column	Transcolumnar Transacetabulum with posterior wall
Anterior wall	Transcolumnar Transacetabulum with anterior wall (ACPH)
Anterior column	Transcolumnar Transacetabulum through the obturator ring
Posterior column with posterior wall	Transcolumnar Supra acetabulum with loss of continuity with axial skeleton
Anterior column with anterior wall	

**Table III:** The new terminology in the proposed classification

New terminology	Old terminology
Transcolumnar transacetabular	Transverse
Transcolumnar transacetabular with posterior wall	Transverse with posterior wall
Transcolumnar transacetabular with anterior wall	ACPH
Transcolumnar transacetabular through the obturator ring	"T"
Transcolumnar supra-acetabular with loss of continuity with axial skeleton	ABC

## Conclusion

In summary, a new method for classification of acetabulum fractures is proposed. It places posterior column with posterior wall fractures to simple fractures instead of complex fractures as in Judet and Letournel classification. The proposed new classification renames “Transverse Fracture” as “Transcolumnar Fracture” to better represent anatomical structure rather than morphology. It is expected that by coining the new term “Transcolumnar”, it will be easy to comprehend the classification of acetabular fractures. It will especially address the confusion between “transverse” and “both column fractures” and would not be a concern in future.
